# Test–Retest Reliability of Repetitive Ocular Vestibular Evoked Myogenic Potentials in Myasthenia Gravis Patients and Healthy Control Subjects

**DOI:** 10.1097/WNP.0000000000000956

**Published:** 2022-06-29

**Authors:** Kevin R. Keene, Julia Bongers, Robert H. P. de Meel, Jeroen Venhovens, Jan J. G. M. Verschuuren, Martijn R. Tannemaat

**Affiliations:** * Department of Neurology and Clinical Neurophysiology, Leiden University Medical Center, Leiden, the Netherlands; and; † Department of Neurology and Clinical Neurophysiology, Albert Schweitzer Hospital, Dordrecht, the Netherlands.

**Keywords:** Myasthenia gravis, Repetitive ocular vestibular evoked myogenic potentials, Test–retest reliability, Reproducibility

## Abstract

**Purpose:**

Repetitive ocular vestibular evoked myogenic potentials (ROVEMP) are a novel diagnostic test to quantify neuromuscular transmission deficits in extraocular muscles in myasthenia gravis. We aimed to investigate the test–retest reliability of the ROVEMP and the effect of amplitude and age.

**Methods:**

We performed the ROVEMP test twice in 19 patients with myasthenia gravis (52.7 ± 19.8 years) and in 15 healthy control subjects (46.5 ± 16 years). The Bland–Altman level of agreement was determined. The relationship between test–retest reliability and signal quality, participant age and signal amplitude was studied.

**Results:**

Limits of agreement were from −179.9 to 139.3 in myasthenia gravis patients and from −56.9 to 89.5 in healthy control subjects. Difference between measurements correlated with signal amplitude (*r* = −0.50, *P* < 0.001). Combining the primary cohort with previously published data from 114 subjects, we found a significant negative correlation between age and reference amplitude (*r* = −0.163, *P* = 0.045).

**Conclusions:**

This study shows that in our hands, the test–retest reliability of the ROVEMP is not optimal. Measurements with higher reference amplitude had a better quality, higher reproducibility, and increased diagnostic yield. We caution against the use of ROVEMP measurements of lower amplitude in clinical practice. In addition, given the correlation between age and amplitude, age matching of healthy control subjects and patients is essential in future studies.

Myasthenia gravis (MG) is an autoimmune disorder affecting neuromuscular transmission, characterized by fluctuating muscle weakness involving variable combinations of ocular, bulbar, limb, and respiratory muscles.^1^ At the onset of the disease, 85% of all patients present with pure ocular symptoms.^2,3^ Early and accurate diagnosis in ocular MG (OMG) is challenging.^2,4,5^ Myasthenia gravis is diagnosed by a combination of typical clinical findings of fatigable muscle weakness and ancillary tests including antibody assays, repetitive nerve stimulation (RNS), single-fiber electromyography (SF-EMG), and edrophonium or neostigmine tests. Unfortunately, in patients with pure ocular symptoms, some of these tests, such as antibody tests and RNS have a low sensitivity,^6^ whereas others, including edrophonium and SF-EMG, are often unavailable outside specialized centers.^7^ Moreover, the edrophonium test has safety concerns and has been primarily studied for ptosis rather than for diplopia in ocular patients.

A growing body of literature suggests that it is possible to quantify neuromuscular transmission deficits in the extraocular muscles (EOMs) using repetitive ocular vestibular evoked myogenic potentials (ROVEMP).^8,9^ The oVEMP reflex is generated by stimulation of the otolith end-organs in response to bone-conducted vibration applied to the skull or air-conducted sound to the ear.^10^ The excitatory myogenic response of the EOMs can be measured with electrodes underneath both eyes.^11,12^ Valko et al.^9^ showed that by applying a train of 10 repetitive oVEMPs, the amplitude of the evoked potential decreases gradually in MG patients, analogous to the decrement observed with RNS. We have previously confirmed this finding in a larger cohort and confirmed the diagnostic yield of this test when patients with other neuromuscular diseases and Graves' orbitopathy were taken as a control group.^8^

Although the ROVEMP seems to be a promising novel diagnostic test in MG, its test–retest reliability has not been studied yet. In previous work, methodologic challenges that influence the signal were addressed: the signal must be averaged 30 times^9^ or 40 times^8^ because of low signal-to-noise ratio, and excessive eye blinking artefacts led to the exclusion of five participants in the work of de Meel et al.^8^ and four participants in the work of Valko et al.^9^ These technical challenges could be a source of variation, potentially impairing the clinical utility of this test in individual patients. Therefore, we aimed to investigate the test–retest reliability of the ROVEMP test in MG patients and healthy control subjects. In addition, we aimed to identify areas amenable to technical refinement by studying the effect of amplitude, electrode resistance, and age on test–retest reliability.

## METHODS

The study was designed as a prospective case–control study, primarily aimed at quantifying the test–retest reliability of the ROVEMP test in MG patients and healthy control subjects in the primary cohort. Previously published data from 114 subjects^8^ was added to increase the power to study the correlation between amplitude and age and the influence of amplitude selection on the diagnostic yield.

### Study Participants

This study was conducted at the outpatient clinic of Leiden University Medical Center between August and November 2020. The Medical Ethical Review Committee of Leiden University Medical Center approved the protocol (NL65522.058.18—P18.091). We obtained written informed consent from all participants. Myasthenia gravis patients were recruited from the Neurology outpatient clinic and healthy control subjects via posters and flyers in the hospital. In 19 MG patients and 15 healthy control subjects, the ROVEMP test was performed twice by the same examiner, with approximately 3 hours between measurements.

The diagnosis of MG was based on typical clinical features, in combination with the presence of serum autoantibodies to the acetylcholine receptor (AChR) or a decrement >10% during RNS. To limit the effect of potential diagnostic uncertainty, seronegative ocular patients without a decrement >10% during RNS were not included. For all participants, we recorded age and sex. For MG patients, we recorded the presence of ptosis, diplopia, and AChR antibodies, presence and size of the decrement found during RNS, Quantitative Myasthenia Gravis score (QMG), and Myasthenia Gravis Activities of Daily Living Profile (MG-ADL). For further analysis of correlations between age, amplitude and diagnostic yield, we combined data from the participants and measurements of the current cohort with those of de Meel et al.^8^

### ROVEMP Test Procedure

**FIG. 1.**
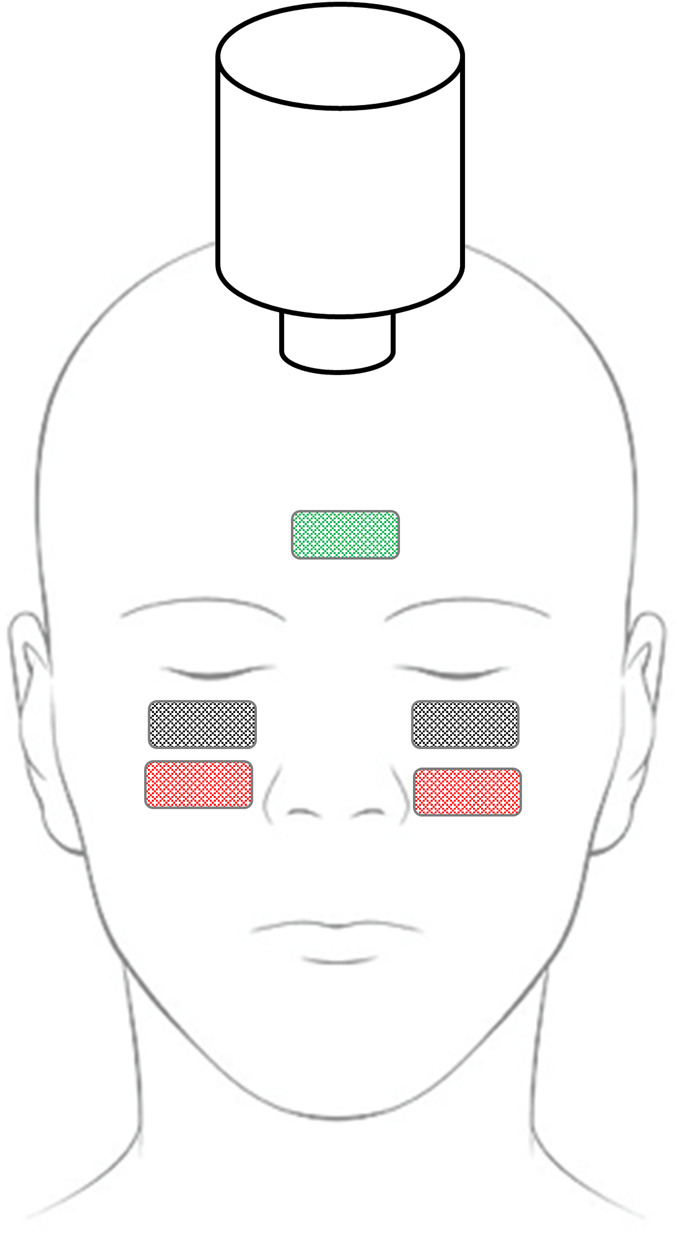
Experimental setup. The stimulus was a bone-conducted skull vibration delivered with a hand-held “mini-shaker” positioned over the hairline. Active (black) and reference (red) electrodes measure the surface EMG signal from the inferior oblique muscles; ground electrode (green).

The procedures were the same as described previously.^8^ Participants were positioned in supine position with their head on a pillow. The skin was cleaned with abrasive gel to improve skin conductance. Active electrodes (black squares in Fig. 1) were placed under each eye while the patient held maximal up-gaze to record myogenic activity from the inferior oblique muscles.^13^ Reference electrodes (red squares in Fig. 1) were placed 2 cm below the active electrodes. The ground electrode was placed on the forehead (Fig. 1). If necessary, the resistance of all electrodes was optimized by repeated application of abrasive gel until an impedance level below 10 kOhm was recorded.

The stimulus was a bone-conducted skull vibration delivered with a hand-held “mini-shaker” positioned at the hairline (minishaker 4810; amplifier 2706, Bruel and Kjaer, Naerum, Denmark). Forty trains of 10 stimuli were administered at a rate of 20 Hz.^14^ The trains were separated by 5 seconds during which participants were asked to close their eyes. The signals were sampled with a rate of 2,000 per second and saved with Nim Eclipse software and a Nim Eclipse recording device (Medtronic Xomed, Inc, Jacksonville, FL). To reduce blinking artefacts, five “practice trains” were administered at the start of the measurement to allow patients to get used to the vibration. Participants were asked to try to suppress blinking during the test. Patients with MG using pyridostigmine were asked to refrain from taking pyridostigmine before the ROVEMP test. To determine reproducibility, the second ROVEMP test was performed approximately 3 hours after the first test by the same examiner.

### ROVEMP Data Postprocessing

**FIG. 2.**
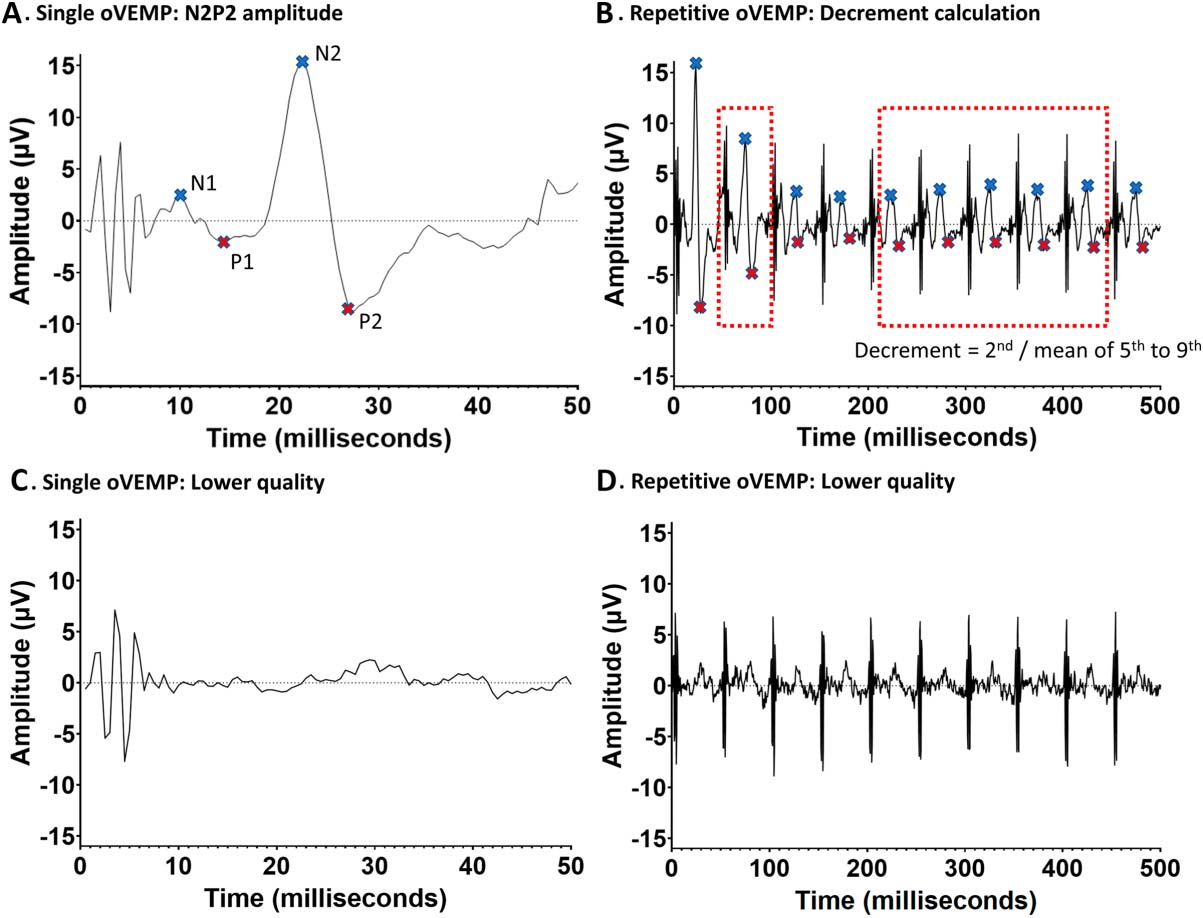
**A**, A high-quality single oVEMP signal, the N1, P1, N2, and P2 are depicted in the figure. The N2P2 amplitude is used as an outcome measure and in decrement calculations. **B**, Example of a repetitive oVEMP train from an MG patient with a clear decremental oVEMP response. Decrement is calculated by dividing the second N2P2 amplitude by the mean of the 5th to 9th amplitude. **C**, An example of a lower quality individual oVEMP with a lower amplitude. **D**, An example of a lower quality repetitive oVEMP train.

Data analysis was performed using an in-house developed MatLab script (MathWorks, Natick, MA), similar to previously described.^8^ All signals were filtered using a 50-Hz notch filter. Single measurement were analyzed for outliers using a median absolute deviation algorithm (MAD) and rejected if the MAD deviated two SDs from the mean of all MADs. Furthermore, an individual measurement was excluded by visual inspection if the signal showed clear noise artefacts. The remaining recorded signals were averaged and filtered using a fourth-order Butterworth 20-Hz high-pass filter to reduce baseline flutter. From the average signal, all peaks and troughs were automatically detected and the relevant oVEMP peaks (N1 and N2, Fig. 2A) and troughs (P1 and P2, Fig. 2A) were selected by the investigator. A peak or trough was automatically detected if there was a minimal increase or decrease of 0.8 µV. The N2P2 amplitude was calculated for each oVEMP potential in the train of 10 stimuli. For readability, the second N2P2 amplitude will be referred to as the reference amplitude throughout the manuscript. Decrement was calculated by dividing the reference amplitude of the ROVEMP signal (Fig. 2A) by the mean of the fifth to the ninth N2P2 amplitude (Fig. 2B).^8,9^

To assess whether the subjective quality of the measurement was related to objective reproducibility and measures such as signal amplitude, three observers (K.R.K., J.B., and M.R.T.) assessed the quality of each single ROVEMP as follows: The quality of a ROVEMP was considered to be high when 10 clear multiphasic waves were observable. Repetitive ocular vestibular evoked myogenic potentials measurements with no clear multiphasic waves or a considerable amount of noise were classified as low quality. Example of a higher quality measurement can be seen in Figures. 2A and 2B and an example of a lower quality measurement can be seen in Figures 2C and 2D. Discrepancies between the assessments of different investigators were resolved by majority vote.

### Statistical Analysis

Statistical analysis was performed using SPSS version 24 (IBM Corp, Armonk, NY). Values of *P* < 0.05 were considered statistically significant. Of the two decrements found in both eyes of the participant, the highest decrement was used for further analysis. The test–retest reliability of the decrement was assessed using Bland–Altman limits of agreement and an intraclass correlation coefficient was calculated. To assess the difference in the reference amplitude between either higher quality or lower quality measurements, as well as MG patients and healthy control subjects, a two-way ANOVA was used. To correlate the reference amplitude with reproducibility, we used the Spearman rank coefficient and used test–retest difference as a surrogate marker for test–retest reliability. To assess whether age affected the reference amplitude, the raw data of de Meel et al.^8^ was added and a Spearman correlation test was performed. To investigate the effect of the amplitude of the ROVEMP signal on diagnostic yield, receiver operating characteristics curve analysis was performed before and after selection of measurements with a reference amplitude of at least 9 µV. For subjects with more than one ROVEMP test, we used the amplitude of the first measurement.

## RESULTS

### Participants

We included 19 patients with MG and 15 healthy control subjects in the primary cohort. The mean age and percentage of men were comparable among patients with MG (52.7 ± 19.8 years; 37%) and healthy control subjects (46.5 ± 16 years; 47%). Demographic and clinical baseline characteristics of all participants are shown in Table 1. The cohort of de Meel et al., used to enrich our amplitude analysis to form a combined cohort, consisted of 92 MG patients (57 ± 18 years old; 48% male) and 22 healthy control subjects (51 ± 14 years; 46% male).

**TABLE 1.** Baseline Characteristics of Myasthenia Gravis Patients and Healthy Controls

Characteristic	Group; Mean (SD)	
	MG (*N* = 19)	Control (*N* = 15)
Age, years	52.7 (19.8)	46.5 (16)
Sex (% male)	37	47
Ptosis (yes)	8	—
Diplopia (yes)	5	—
AChR+ (positive)	17	—
Decrement > 10% RNS	7	—
QMG	12.9 (6.2)	—
MG-ADL	7.2 (3.6)	—

MG-ADL, Myasthenia Gravis Activities of Daily Living score; QMG, Quantitative Myasthenia Gravis score.

### Test–Retest Reliability

**FIG. 3.**
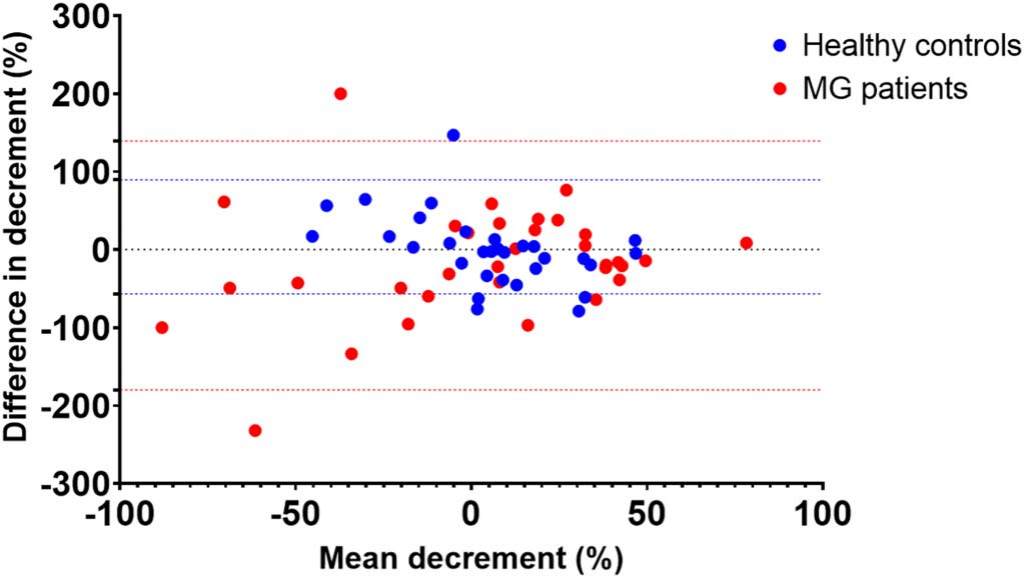
Bland–Altman plot for the test and retest reliability separated for healthy controls (in blue) and myasthenia gravis patients (in red) per eye. The limits of agreement of MG patients were from −179.9 to 139.3, and the limits of agreement of healthy controls were from −56.9 to 89.5.

The limits of agreement of MG patients were from −179.9 to 139.3, and the limits of agreement of healthy control subjects were from −56.9 to 89.5 in the primary cohort (Bland–Altman in Fig. 3). Myasthenia gravis patients had a negative bias of −18.8%, whereas healthy control subjects showed a small positive bias of 3.1%. The intraclass correlation coefficient for the intrarater reliability was poor: −0.08 (−0.40 to 0.26) for MG patients and 0.03 (0.33–0.39) for healthy control subjects.

### Reference Amplitude

Simple main effects analysis showed that measurements classified as “high quality” had a significantly higher reference amplitude (i.e., the second N2P2 amplitude, used in decrement calculations) (9.58 ± 6.03 µV) than measurements of lower quality (5.51 ± 4.71 µV, *P* < 0.001). In addition, control subjects had a significantly higher reference amplitude (9.49 ± 6.47 µV) than MG patients (5.47 ± 4.15 µV, *P* = 0.001) in the primary cohort. A two-way ANOVA revealed a significant interaction effect between group and quality score on the reference amplitude, meaning that the influence of amplitude on quality of the measurement is different for MG patients and healthy control subjects (*P* = 0.015).

**FIG. 4.**
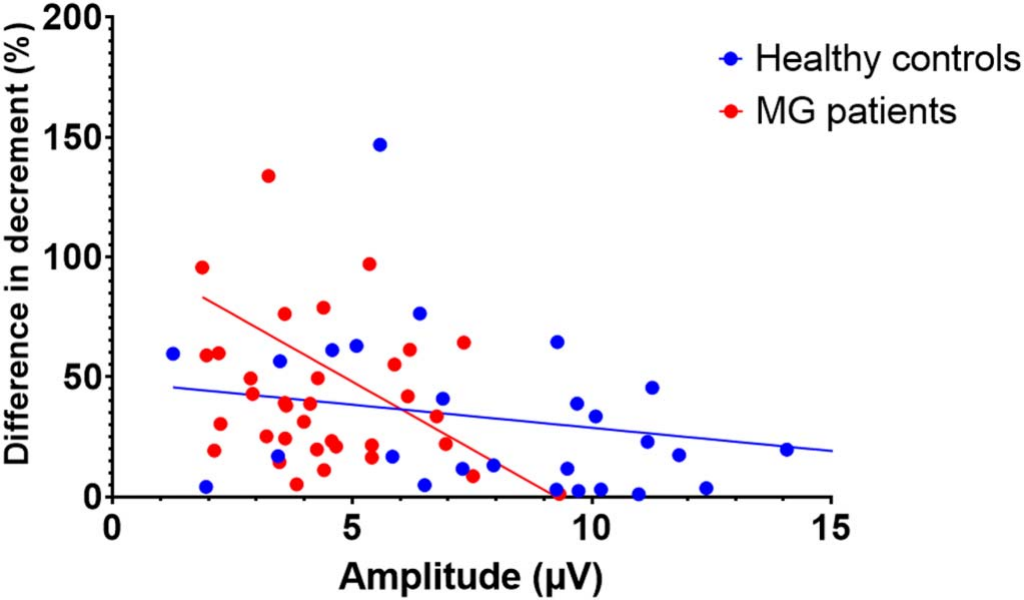
Correlations between the difference in decrement between the test and retest measurement, as a proxy for reproducibility, and the reference N2P2 amplitude. Subjects with higher amplitudes seem to have a higher test–retest reliability because the difference in decrement is significantly lower. Correlations and individual values are shown for healthy controls (blue) and myasthenia gravis patients (in red).

A significant negative correlation was observed between the difference in decrement of the first and second measurements, used here as a proxy for reproducibility, and the reference amplitude (*r* = −0.50, *P* < 0.001, Fig. 4), suggesting that reproducibility was correlated with the amplitude of the signal.

### Influence of Electrode Resistance

In the primary cohort, the mean measured electrode resistance was 5.26 ± 1.7 kOhm. The resistance did not differ between the higher quality (5.36 ± 1.96 kOhm) and lower quality (5.63 ± 1.53 kOhm) measurements (*P* = 0.40). Moreover, there was no correlation between electrode resistance and the reference amplitude (*r* = 0.05, *P* = 0.55).

### Influence of age

**FIG. 5.**
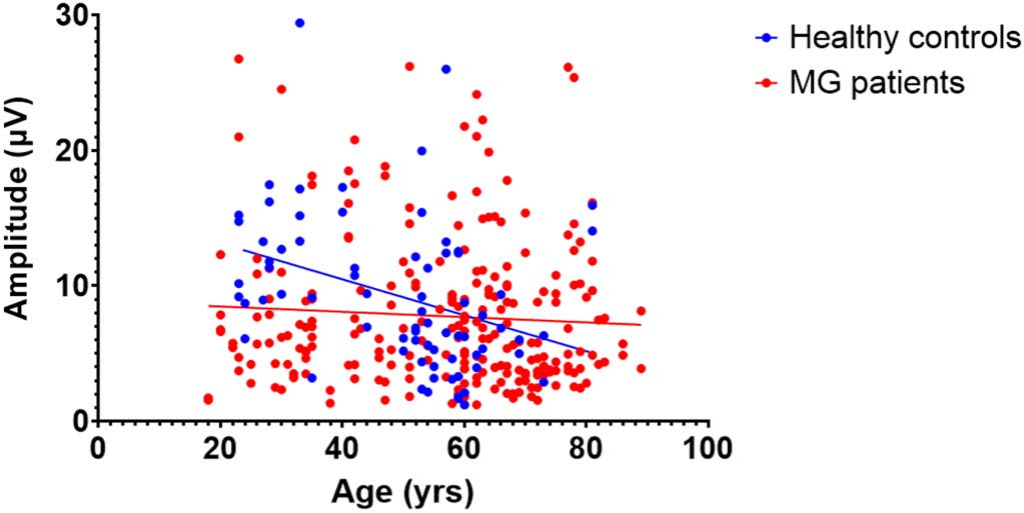
For healthy controls (blue) and MG patients (red), the correlation between age and reference amplitude is shown. Healthy controls showed a significant negative correlation between age and reference amplitude (*r* = −0.42, *P* = 0.009). However, for MG patients, no significant correlation was found (*r* = −0.11, *P* = 0.240).

Patients with high-quality measurements were younger, as demonstrated by the significant difference in age for high-quality (40.3 ± 15.6 years) and low-quality (58.9 ± 15.1 years) measurements (*P* < 0.001). In the primary cohort, we found a significant correlation between age and amplitude in the first measurements (*r* = −0.282, *P* = 0.024), which was mainly found in the healthy control subjects (*r* = −0.437, *P* = 0.16) and not in the MG patients (*r* = −0.033, *P* = 0.852). When we combined data from the 34 subjects studied in this work with previously published data from 114 subjects,^8^ we also found a significant negative correlation between age and the reference amplitude in the combined cohort (*r* = −0.163, *P* = 0.045) (Fig. 5). This correlation was driven by healthy control subjects, in whom we observed a significant negative correlation between age and reference amplitude (*r* = −0.42, *P* = 0.009). In contrast, for MG patients, no significant correlation was found (*r* = −0.11, *P* = 0.240).

### Diagnostic Yield and Reference Amplitude

**FIG. 6.**
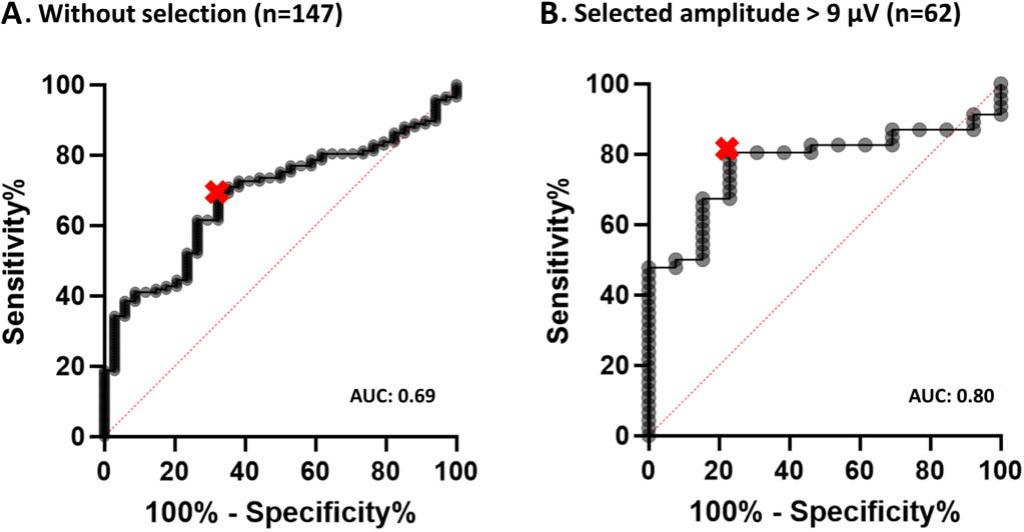
Receiver operating characteristics analysis showing the effect of selecting only measurements with high amplitudes on diagnostic yield. **A**, Entire cohort: optimal cutoff value >15.81%, sensitivity 68%, specificity 71%, and area under the curve (AUC) 0.69. **B**, After selecting of cases with a reference N2P2 amplitude of at least 9 µV. Optimal cutoff value >11.26%, sensitivity 81%, specificity 81%, and AUC 0.80.

Receiver operating characteristics analysis based on the combined cohort of 147 subjects (37 healthy control subjects and 111 MG patients) showed an area under the curve of 0.69 (sensitivity of 68% and specificity of 71% at a cutoff of 16% decrement). Diagnostic yield improved substantially when only measurements with a reference amplitude of at least 9 μV were included (16 healthy control subjects and 46 MG patients): AUC was 0.80 and both sensitivity and specificity increased to 81% at a cutoff value of 11.26% (Fig. 6). Additionally, receiver operating characteristics analysis on participants below the age of 55 years (the mean of the combined cohort) showed an AUC of 0.73 as compared with an AUC of 0.54 for participants above the age of 55 years, confirming the influence of age on diagnostic yield.

## DISCUSSION

We aimed to study the test–retest reliability of the ROVEMP test for diagnosing myasthenia gravis and identify areas amenable to technical refinement. For MG patients, Bland–Altman analysis showed limits of agreement from −179.9% to 139.3%. In healthy control subjects, the limits of agreement were smaller (−56.9% to 89.5%). In the context of previously proposed cutoff values of 9% and 15%^8,9^ for MG diagnosis, the test–retest reliability of the ROVEMP is suboptimal.

Several observations indicate that the reproducibility of the ROVEMP is related to its signal-to-noise ratio (SNR) and that the amplitude of the signal (expressed here as the amplitude of the reference N2P2) is the limiting factor for many measurements. The N2P2 amplitude was significantly correlated with the absolute difference between two repeated tests and the diagnostic yield improved when only measurements with a reference amplitude of at least 9 μV were included. In addition, measurements judged to be of low quality by qualitative visual assessment had significantly lower amplitudes than measurements of high quality. Unfortunately, the amplitude of the signal appears to be affected by disease, as MG patients had a significantly lower second N2P2 amplitude than healthy control subjects, and age, with higher amplitudes in younger participants. Surprisingly, the measured electrode resistance did not correlate with the amplitude of the signal, suggesting that technical optimization by reducing electrode resistance is not a viable method to improve SNR. Not all variability in amplitude between subjects can be explained by these factors; the cause is currently unclear.

We hypothesize that the lower amplitudes observed in MG patients are related to the disease, analogous to the previously described finding that compound muscle action potential (CMAP) amplitudes are lower in MG patients with severe disease.^15,16^ Moreover, the amplitude of the reference amplitude declined significantly with age in healthy control subjects. This corresponds to previous research showing lower N1P1 oVEMP amplitudes in older adults^10^ and lower CMAP amplitudes in a healthy older adults.^17^ Unfortunately, these findings might reduce the clinical utility of the ROVEMP. The average age at onset of MG is 44 years,^18^ and because the subjective quality of the ROVEMP signal and objective measures such as the N2P2 amplitude decline with age, the quality of a measurement must be taken into account for older patients in clinical practice because diagnostic yield seems to be low in subjects older than 55 years. In previous studies, more young healthy control subjects were included compared with the patient groups: in the work of Valko et al.,^9^ the mean age was 47 years for healthy control subjects and 58 years for patients, and in the work of de Meel et al.,^8^ the SD of the age was higher in the healthy control group. Moreover, the number of ocular MG patients was slightly higher in these studies compared with the current work. Both the difference in age and phenotype may help explain the higher AUC found in these studies, respectively, 0.80 and 0.78, compared with the AUC of 0.69 in the current work. In addition, in the work of Valko et al., 4 subjects (7% of the total population) were excluded from further analysis because of excessive blinking artifacts. Similarly, the results of five subjects were excluded by de Meel et al. because of excessive artefacts. The exclusion of these measurements in both studies may have skewed the results toward a higher diagnostic yield compared with the current study. In future diagnostic studies, age matching between healthy subjects and MG patients must be performed to avoid bias as a result of age effects on the ROVEMP signal. Additionally, future studies including a younger cohort of healthy control subjects and MG patients prospectively could potentially show a higher diagnostic yield because young subjects are likely to have higher amplitudes and more reliable tests.

Limitations of this study include the single center of inclusion and the single observer performing the tests and retests. However, it is likely that reproducibility would have decreased further when data from different centers would have been combined. Our main conclusions would therefore probably not be affected.

Measurements were selected using the arbitrary cutoff of 9 μV. Although this threshold was chosen somewhat arbitrarily, it confirmed the negative effect of the signal amplitude on clinical utility of the ROVEMP by comparing the diagnostic yield of the entire cohort to a selection of cases with a higher reference amplitude, or a lower age, and showing that the AUC, sensitivity, and specificity were all higher when cases with a low amplitude were excluded.

Despite its low reproducibility, our results confirm that the ROVEMP seems to be able to detect a decrement in patients with MG, suggesting that it is able to quantify neuromuscular transmission deficits in the EOMs. This is highly promising because there is no alternative neurophysiological method to study the EOMs, which are the most commonly affected muscles in MG.

However, we caution against the use of ROVEMP measurements of lower amplitude in clinical practice. In diagnostically challenging patients, a ROVEMP test result with a high amplitude and clear decrement could be of clinical value in addition to standard diagnostic tests. Unfortunately, this approach limits the use of this test in a substantial number of patients because only 42% of all subjects in our investigations had a reference amplitude of at least 9 μV. Particularly in older patients, it is likely that the ROVEMP will not yield an unequivocal result. Based on our current findings, we believe that the ROVEMP test is especially suitable to detect changes at group level (e.g., in the context of studies on therapeutic interventions).

## CONCLUSIONS

This study shows that the test–retest reliability of the ROVEMP is not optimal. Measurements with a higher reference amplitude had a better quality, higher reproducibility, and an increased diagnostic yield. Therefore, we caution against the use of ROVEMP measurements of lower amplitude in clinical practice. We recommend further studies aimed at optimization of the SNR to increase diagnostic yield and reliability of the ROVEMP, perhaps by improving stimulus parameters^14^ or reducing blink artefacts. In addition, given the correlation between age and amplitude, age matching of healthy control subjects and patients is essential in future studies.
